# Abundance and Survival Rates of the Hawai’i Island Associated Spinner Dolphin (*Stenella longirostris*) Stock

**DOI:** 10.1371/journal.pone.0086132

**Published:** 2014-01-22

**Authors:** Julian A. Tyne, Kenneth H. Pollock, David W. Johnston, Lars Bejder

**Affiliations:** 1 Murdoch University Cetacean Research Unit, Centre for Fish, Fisheries and Aquatic Ecosystems Research, School of Veterinary and Life Sciences, Murdoch University, South Street, Murdoch, Western Australia, Australia; 2 Department of Biology, North Carolina State University, Raleigh, North Carolina, United States of America; 3 Division of Marine Science and Conservation, Nicholas School of the Environment, Duke University Marine Lab, Beaufort, North Carolina, United States of America; Dauphin Island Sea Lab; University of South Alabama, United States of America

## Abstract

Reliable population estimates are critical to implement effective management strategies. The Hawai’i Island spinner dolphin (*Stenella longirostris*) is a genetically distinct stock that displays a rigid daily behavioural pattern, foraging offshore at night and resting in sheltered bays during the day. Consequently, they are exposed to frequent human interactions and disturbance. We estimated population parameters of this spinner dolphin stock using a systematic sampling design and capture–recapture models. From September 2010 to August 2011, boat-based photo-identification surveys were undertaken monthly over 132 days (>1,150 hours of effort; >100,000 dorsal fin images) in the four main resting bays along the Kona Coast, Hawai’i Island. All images were graded according to photographic quality and distinctiveness. Over 32,000 images were included in the analyses, from which 607 distinctive individuals were catalogued and 214 were highly distinctive. Two independent estimates of the proportion of highly distinctive individuals in the population were not significantly different (*p* = 0.68). Individual heterogeneity and time variation in capture probabilities were strongly indicated for these data; therefore capture–recapture models allowing for these variations were used. The estimated annual apparent survival rate (product of true survival and permanent emigration) was 0.97 SE±0.05. Open and closed capture–recapture models for the highly distinctive individuals photographed at least once each month produced similar abundance estimates. An estimate of 221±4.3 SE highly distinctive spinner dolphins, resulted in a total abundance of 631±60.1 SE, (95% CI 524–761) spinner dolphins in the Hawai’i Island stock, which is lower than previous estimates. When this abundance estimate is considered alongside the rigid daily behavioural pattern, genetic distinctiveness, and the ease of human access to spinner dolphins in their preferred resting habitats, this Hawai’i Island stock is likely more vulnerable to negative impacts from human disturbance than previously believed.

## Introduction

Many islands in tropical and sub-tropical regions represent isolated oases of marine life, exhibiting higher levels of primary productivity, secondary productivity and enhanced communities of top predators than the oligotrophic pelagic background around the islands [1]. In many situations, the cetacean top predators that have evolved to exploit island-associated productivity in these regions represent resident, isolated populations, often with high site fidelity and restricted gene flow amongst nearby island regions [2]–[4]. Furthermore, many island associated small cetacean populations exhibit specialized behaviours and social dynamics that have evolved to facilitate their survival. However, due to their specialized demography and behavioural ecology, it is becoming increasingly clear that island-associated, populations of small odontocetes may be particularly vulnerable to anthropogenic effects (e.g. false killer whales in the Hawaiian Archipelago). Hawaiian spinner dolphins represent one such species – they exist as small isolated populations with restricted ranges and exhibit a specialized behavioural ecology [5], [6] that renders them vulnerable to human activities in coastal environments. Spinner dolphins occur in sub-tropical and tropical oceans worldwide and are named because of their aerial behaviours [7]. Gray’s spinner dolphin, (*Stenella longirostris*), is the most widely distributed subspecies [8] and occurs throughout the entire Hawaiian archipelago.

The Hawaiian archipelago consists of the mainly uninhabited North West Hawaiian Islands, from Kure Atoll in the north to the eight inhabited main Hawaiian Islands, with Hawai’i Island in the south. Recent genetic analyses revealed that Hawaiian spinner dolphins are distinct from populations found elsewhere [9], and moreover, subpopulations within the Hawaiian archipelago were also found to be genetically distinct [10]. As a consequence, Hawaiian spinner dolphins have been divided into five different island/island-group *management units* under the US Marine Mammal Protection Act (MMPA) by the National Oceanic and Atmospheric Administration’s National Marine Fisheries Service (NMFS) that correspond with two broad geographical regions: 1.) three in the Main Hawaiian Islands: Hawai’i Island, Oahu/4-Islands area, Kauai/Niihau, and 2.) two in the Northwest Hawaiian Islands: Pearl & Hermes Reef and Kure/Midway. The NMFS is mandated by the MMPA to assess the population status and threats for all identified stocks of marine mammals in U.S. waters.

At present, reliable abundance estimates are not available for any stock of Hawaiian spinner dolphins, a significant impediment to developing appropriate management plans for any spinner dolphin management unit in Hawai’i. Previously, a line transect survey of the entire Hawaiian Exclusive Economic Zone resulted in an abundance estimate of 3,351 [11] spinner dolphins throughout the entire Hawaiian archipelago which assumed a single Hawaiian stock [12]. Considering the ship’s track and the coastal daytime reliance of this species, the large ship-based estimate provided by [11] is not appropriate for estimating the abundance of inshore spinner dolphins. Other studies which estimated the abundance of spinner dolphins along the Kona Coast were based on opportunistic photo-identification sightings and were not specifically designed to estimate abundance [6], [13]–[15]. As a consequence, a collaborative project, ‘*spinner dolphin acoustics population parameters and human interaction research*’ (SAPPHIRE) was developed in 2010 to assess the abundance, distribution and behaviour of spinner dolphins along the Kona Coast. The SAPPHIRE project combines boat-based photo-identification and group focal follows and land-based theodolite observations, along with passive acoustic monitoring, to evaluate the effects of human interactions on spinner dolphins in the region.

Hawaiian spinner dolphins exhibit a rigid, diurnal behavioural pattern. At night, they forage cooperatively offshore in deeper water [16]. During the day, they move into shallow, coastal habitats to rest and socialise [5], preferring sandy-substrate locations that are sheltered from the wind, typically less than 50 m deep (possibly to aid in predator detection) and within close proximity to their deep-water foraging areas [5], [6], [17]. This rigid, behavioural pattern is unlike the less predictable patterns observed in other coastal dolphin species, such as the bottlenose dolphin (*Tursiops* sp.), a species known to readily switch between behavioural states, e.g. from foraging to resting to socialising [18]. To maintain this rigid behavioural pattern, spinner dolphins are dependent on these sheltered bays to rest [5], [6]. However, within these same habitats, dolphins are easily accessible and thereby exposed to human interactions and disturbance [19]–[22]. When anthropogenic impacts are considered in combination with their genetic distinctiveness and low gene flow, Hawaiian spinner dolphin’s susceptibility to human disturbance is of serious and increasing concern for the survival of the stock.

During periods of activity, animals usually exhibit enhanced brain function, which is often referred to as vigilance. Vigilance is required for many activities including foraging, socializing and predator avoidance. As animals undertake these cognitively challenging activities they tire, and accrue what is often referred to as a vigilance decrement [23]. In higher vertebrates, vigilance decrements can manifest in a decreased ability to detect camouflaged predators or cryptic prey [23]. They may also manifest in more abstract ways such as reduced decision-making capabilities [23]. To recover from a vigilance decrement, animals must rest [24]. The derived behavior of spinner dolphins renders them especially vulnerable to interrupted resting bouts during the day, as they have a limited ability to recover before embarking on another foraging bout the following evening.

Dolphin-watch tourism can cause biologically significant effects on exposed communities by causing habitat displacement [25], [26]. Short-term studies reveal that an increase in vessel, kayak and swimmer traffic both inside and outside of known resting bays in Hawai’i have resulted in spinner dolphins spending less time in important habitats [14]. Consequently, their rest periods are truncated and interrupted [19], [27], [28]. This type of anthropogenic disturbance of spinner dolphins in their resting habitat may have negative, long-term impacts that will likely reduce their distribution and abundance over the long term [14], [29]. Unfortunately, current scientific literature is lacking accurate information to inform how long-term disturbance may impact Hawaiian spinner dolphins, specifically in response to the cumulative exposure of human disturbance in important resting habitats.

For small cetaceans, capture-recapture studies, based on photo-identification, have proven to be a reliable method for estimating population parameters, such as abundance, survival and recruitment rates [30]–[35]. However, the characteristics of individual cetaceans and the methods used to photograph them can introduce heterogeneity in the capture probabilities and misidentification of individuals [36]. Careful attention to the study design can help improve the adherence of the sampling methodology, to the assumptions of capture-recapture models and mitigate biases due to heterogeneity and misidentification of dolphins. Two types of population models are generally considered for capture-recapture sampling designs: closed and open population models [31], [32], [34], [35], [37], [38]. During long-term studies, it is not always possible to assume that the population being studied is closed, and therefore, open population models should be used [30], [31], [35].

As part of the SAPPHIRE research project, the objectives of this study were to estimate the population abundance and survival rate of the Hawai’i Island spinner dolphin stock using a systematic sampling design, and both open and closed capture-recapture population models. The resulting scientific data are the first to provide accurate and reliable baseline population estimates for this stock. This information will be useful for management agencies for both stock assessment purposes, and to assess the effectiveness of planned management actions that are aimed at mitigating negative impacts of human-dolphin interactions.

## Materials and Methods

### Fieldwork

#### Ethics statement

Data were collected under National Oceanographic and Atmospheric Administration permit GA15409 and under approval of the Murdoch University Animal Ethics Committee permit W2331/10.

#### Study area

**Figure 1:**
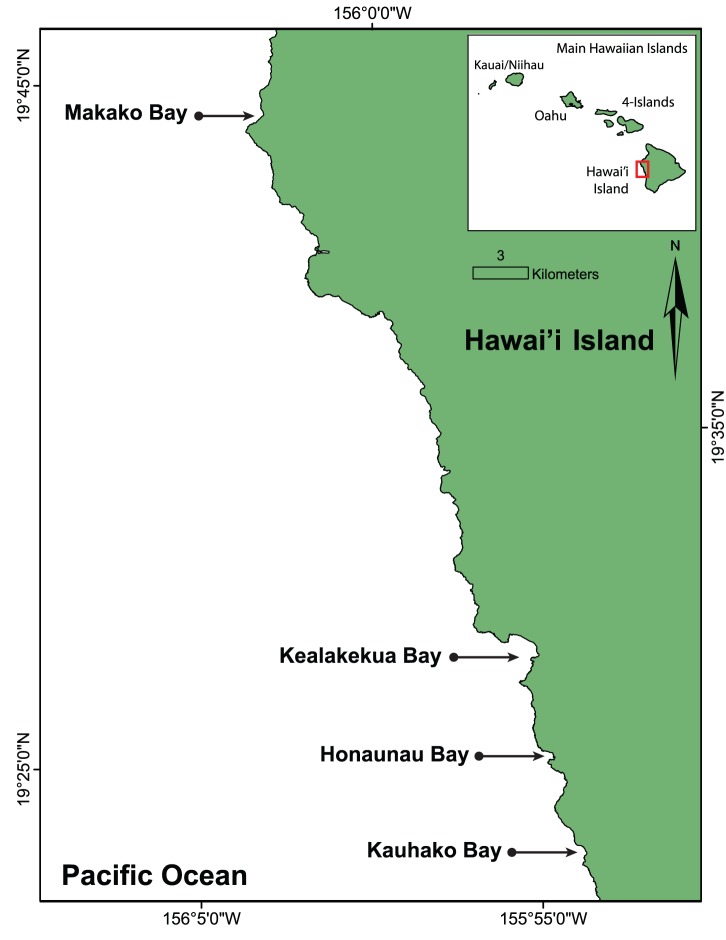
Map of the study area illustrating the locations of the four spinner dolphin resting bays, Kauhako Bay, Honaunau Bay, Kealakekua Bay and Makako Bay, along the Kona Coast of Hawai’i Island in relation to the other island regions in the Main Hawaiian Islands (inset).

The Hawaiian archipelago is located in the Pacific Ocean, approximately 3,200 km southwest of mainland United States. Hawai’i Island is the largest, youngest and most southerly of the main Hawaiian Islands. On the leeward side of the island is the Kona Coast, where the four main spinner dolphin resting bays are located (Figure 1): Kauhako Bay, Honaunau Bay, Kealakekua Bay and Makako Bay [5], [6], [19]. In addition, these bays are consistently used by boats, kayaks, stand-up paddle boards and swimmers for recreational purposes, thus providing opportunities for people to interact with the resting dolphins [19]–[21].

#### Sampling design

A systematic sampling design was developed to study the Hawai’i Island spinner dolphin stock. From September 2010 to August 2011 (excluding May 2011) boat-based photographic-identification surveys were carried out during 12 days of each month in the four resting bays in a sequential order: Kauhako Bay for four days; Honaunau Bay for two days; Kealakekua Bay for four days; and Makako Bay for two days. We would arrive at a bay (only one bay each day) at 0700 h. If the dolphins weren’t present we would wait until 1600 h to see if they would arrive. We carried out boat-based photo-identification (see below) if dolphins were present (or arrived during the day). Each bay was systematically surveyed on the same dates each month, regardless of whether dolphins were present or absent. This sampling regime provided consistent and even effort throughout the study period and area.

#### Photographic-identification

The boat-based photo-identification team consisted of three to five observers with two digital SLR cameras: a Nikon D300s and a Nikon D300, both with Nikon 80 mm to 400 mm AF VR Zoom lenses. We used a ‘100 m chain rule’ [39], [40], to determine members of each group of spinner dolphins, where any animals within 100 m of each other were considered to be members of the same group. When a dolphin group was sighted the dolphins were approached for surveying and group size was determined. With dolphin groups < = 20 we had a greater probability of obtaining good photographs of all individual group members which, in turn optimised the chance that the more distinctly marked individuals were not more likely to be photographed than the less distinctly marked individuals. Photographs were taken when dolphins surfaced within 25 m of the research vessel. A dolphin survey would last a minimum of 30 minutes and maximum an hour with a minimum of a 30 minute break between surveys. Breaks between dolphin group surveys were to limit the disturbance to the focal group from the research vessel. Repeated dolphin group surveys optimised the probability of capturing all animals in the group. Field observations also noted if groups from outside the bays joined the focal group. Dolphin surveys would continue throughout the day until either: the whole group was photographed, the dolphins left the bay, or when environmental conditions deteriorated, i.e. sea state>Beaufort 2.

#### Grading and sorting of photo-identification images

All photographs were graded according to photographic quality and distinctiveness in order to minimise the introduction of bias and to reduce misidentification [31], [41]–[43]. Following [41], all photographs were assigned absolute values based on clarity and focus (2, 4 or 9), degree of contrast (1 or 3), angle of dorsal fin to the camera (1, 2 or 8), dorsal fin visibility and the proportion of the frame filled by the dorsal fin (1 or 5). These values were then summed to produce an overall image quality score. Excellent quality images received scores of 6–7, good quality images had scores from 8–11, and poor quality images had scores >11 [31], [41].

Dorsal fin distinctiveness varies between individual dolphins; thus not all fins were distinctively marked enough to be included in capture-recapture analyses [31], [37], [44]. As a consequence, photographs were analysed for individual distinctiveness based on patterns of nicks and notches on the leading and trailing edges of the dorsal fin that were visible from both sides [41]. Overall distinctiveness was based on a scale of *D1* (highly distinctive, features evident in distant and poor quality photographs), *D2* (smaller less distinctive nicks and notches) and *D3* (not distinctive) [31], [41]. Individuals with a distinctiveness rating of *D1* or *D2* were integrated into the photographic-identification catalogue and highly distinctive individuals (*D1*) were used to calculate the mark rate of the stock which in turn, was used to scale up to estimate total stock size. Every individual was compared to all others in the catalogue before being assigned a unique identification code and added separately to the catalogue. Individuals with a distinctiveness rating of *D3* were given a generic identification code but not included in the catalogue.

### Analyses

#### Capture–recapture

A capture was defined as a photograph of sufficient quality of an individual dolphin’s distinctly marked dorsal fin. Only highly distinctive (*D1*) fins in photographs of excellent and good quality were included in the capture-recapture analyses to reduce misidentification errors. Capture histories corresponded to whether or not an individual was “captured” or “recaptured” during a sampling occasion. This information was compiled for each individual (excluding calves), after the photo grading process. The program MARK [45] contains a suite of capture-recapture models and goodness-of-fit tests. Using MARK open and closed capture-recapture models were then applied to these data.

All capture-recapture models make the following assumptions [46]: 1) marks are not lost during the study; 2) marks are correctly recognised on recapture; 3) individuals are instantly released after being marked; 4) intervals between sampling occasions are longer than the duration of a sample; 5) all individuals observed during a given sampling occasion have the same probability of surviving until the next one; 6) study area does not vary; and 7) homogeneity of capture probabilities, i.e. that all animals in a sampling occasion have equal probability of being captured. This assumption is relaxed for certain models which do allow heterogeneity of capture probabilities.

#### Estimating abundance and demographic parameters

A variety of closed and open capture-recapture models were fitted using the program MARK [45]. They used the capture histories of all highly distinct individuals captured on at least one occasion during each month in any of the four bays. Therefore, the population abundance estimate refers to the highly distinct individuals.

POPAN [47] is an integrated combined likelihood formulation of the original Jolly-Seber open capture-recapture model [48], [49]. POPAN estimates a super-population size (N), entry probabilities, apparent survival rates and capture probabilities. Maximum likelihood was used to estimate the following parameters: N, the super population size, which is all the animals that existed in the population (stock) at any point during the study period; *φ_t_* is the apparent survival probability from sampling period *t* to sampling period *t +1* and is the product of true survival times the probability the animal does not emigrate; *p_t_* is the probability that an individual available for capture in sampling period *t* would be captured in sampling period *t;* and *β_t_* is the probability of entry of an individual into the population between sample *t* and sample *t +1*. Derived estimates of the stock sizes at each sampling time (N_t_) can also be estimated if necessary.

A suite of POPAN candidate models were developed to allow for fixed or time-varying effects on the entry probabilities, apparent survival rates and capture probabilities. For model selection, Akaike’s Information Criterion (AIC) was applied, which provides a measure of the model fit but is penalized when there is an increase in the number of parameters [50]. RELEASE, a goodness-of-fit program in MARK [45], was used to determine goodness-of-fit for the POPAN models [51]. Over-dispersion in the models was accounted for, by estimating the over-dispersion measure ĉ using the chi-square statistic divided by its degrees of freedom. QAIC values were used for model selection [52] with the lowest QAIC value an indication of the most parsimonious model.

MARK was also used to obtain closed population model estimates for models that allow heterogeneity and time variation of capture probabilities (M_0,_ M_h_, M_t_ and M_th_) [45], because spinner dolphins are long lived. The advantage of using the closed models, if appropriate, is that they provide estimates with higher precision than open models and allow for heterogeneity of capture probabilities among individuals, which is very common in most capture-recapture studies [30].

#### Estimation of mark rate and total stock size

Estimates of the stock size from the capture–recapture models relate only to the identifiable animals in the study. Therefore, to estimate the total stock size, estimates need to be scaled based on the proportion of individuals that are identifiable. Here, we estimated the proportion of highly distinctive individuals (*D1*) in the Hawai’i Island spinner dolphin stock using two independent measures of mark rates: 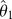 and 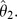


Mark Rate 1 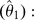 For groups consisting of >20 dolphins, a mark rate was calculated from the proportion of randomly taken photographs that contained identifiable dolphins that were obtained from the two photo-identification cameras that were working simultaneously [37], [53]. To be included in the analyses, photographs had to be of sufficient quality to identify a dolphin if it had been identifiable.
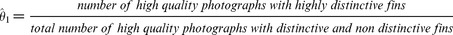



Mark Rate 2 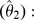 A second independent mark rate was calculated using only the photo-identification data collected for group sizes that were ≤20 dolphins. Unlike with large groups, this scenario assumed that all individuals in the group were photographed to a quality that would allow dolphins to be identified if they were identifiable. Thus, for each group that consisted of ≤20 dolphins, 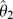 was calculated based on the knowledge of group size, together with the number of highly distinctive individuals in each group:
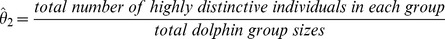



The standard errors (SE) for both mark rate estimates are:
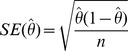
where *n* is the sample size in each equation.

Both of these methods assumed that the proportion of identifiable individuals in the sample was equivalent to the proportion of identifiable individuals in the entire stock [54]. The numbers of highly distinctive and non-distinctive individuals were summed over all surveys and used to estimate the total number of individuals in the stock:
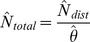



Where 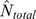 is the estimated abundance of all individuals (distinctive and non-distinctive) identified during the study period, 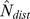 is the abundance estimate of the highly distinctive individuals, and 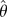 is the estimated proportion of distinctive individuals [55].

The variance for the total stock size estimate was derived as follows [46]:
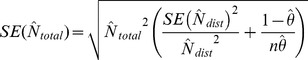



Log-normal 95% confidence intervals were calculated with a lower limit of 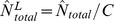 and upper limit of 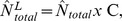 [55] where:
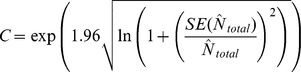



## Results

### Effort and Summary Statistics

From September 2010 to August 2011, photo-identification surveys were carried out for a total of 132 days (>1,150 hours of effort; >100,000 dorsal fin images) in the four bays. More than 32,000 images were of sufficient quality to be added to the catalogue, from which 607, *D1* and *D2* individuals were identified and contained 214 highly distinctive, *D1*, individuals.

**Figure 2:**
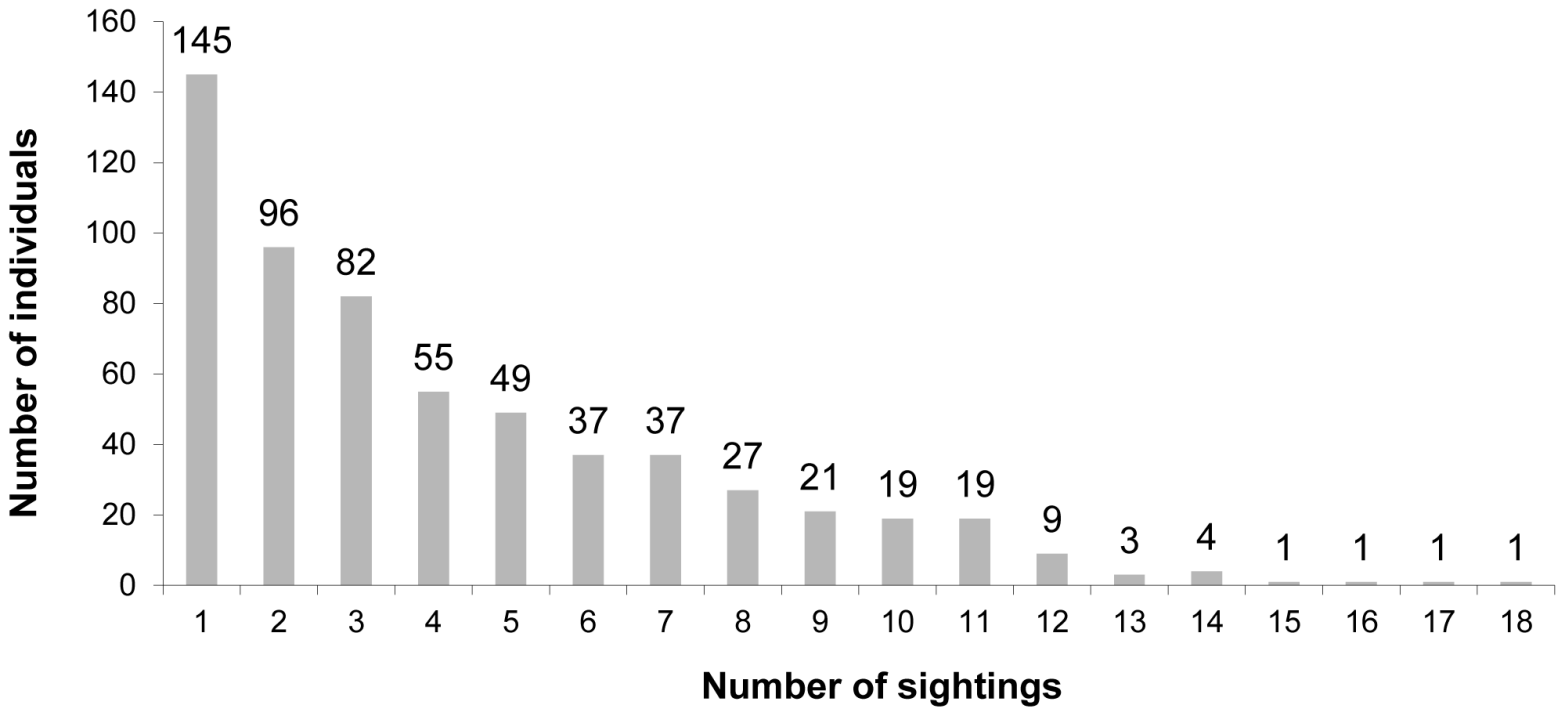
Frequency of individual spinner dolphin sightings from September 2010 to August 2011.

**Figure 3:**
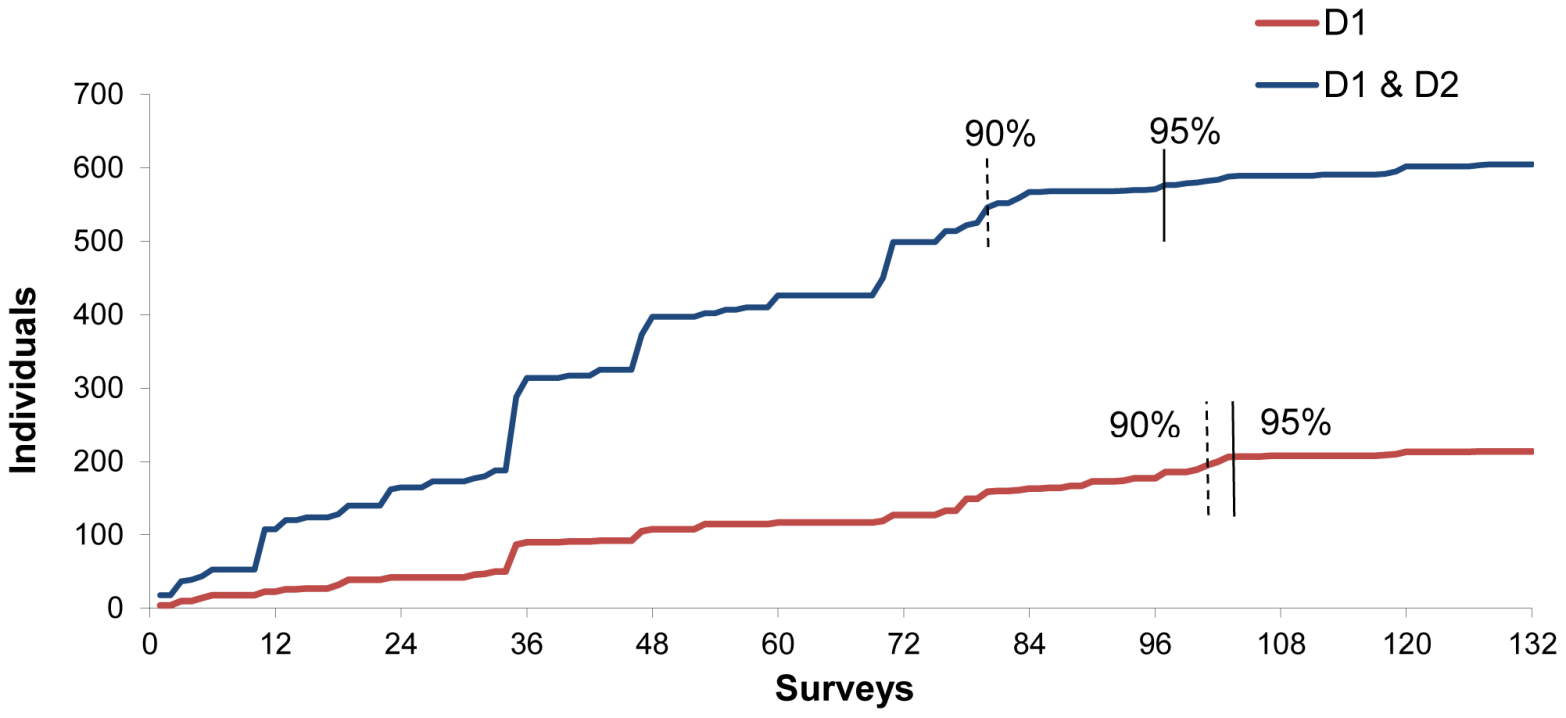
Cumulative discovery curve of highly distinctive (D1) and distinctive (D2) Hawaiian spinner dolphins during 132 photographic identification surveys in the study area (all four bays combined) from September 2010 to August 2011.

**Figure 4:**
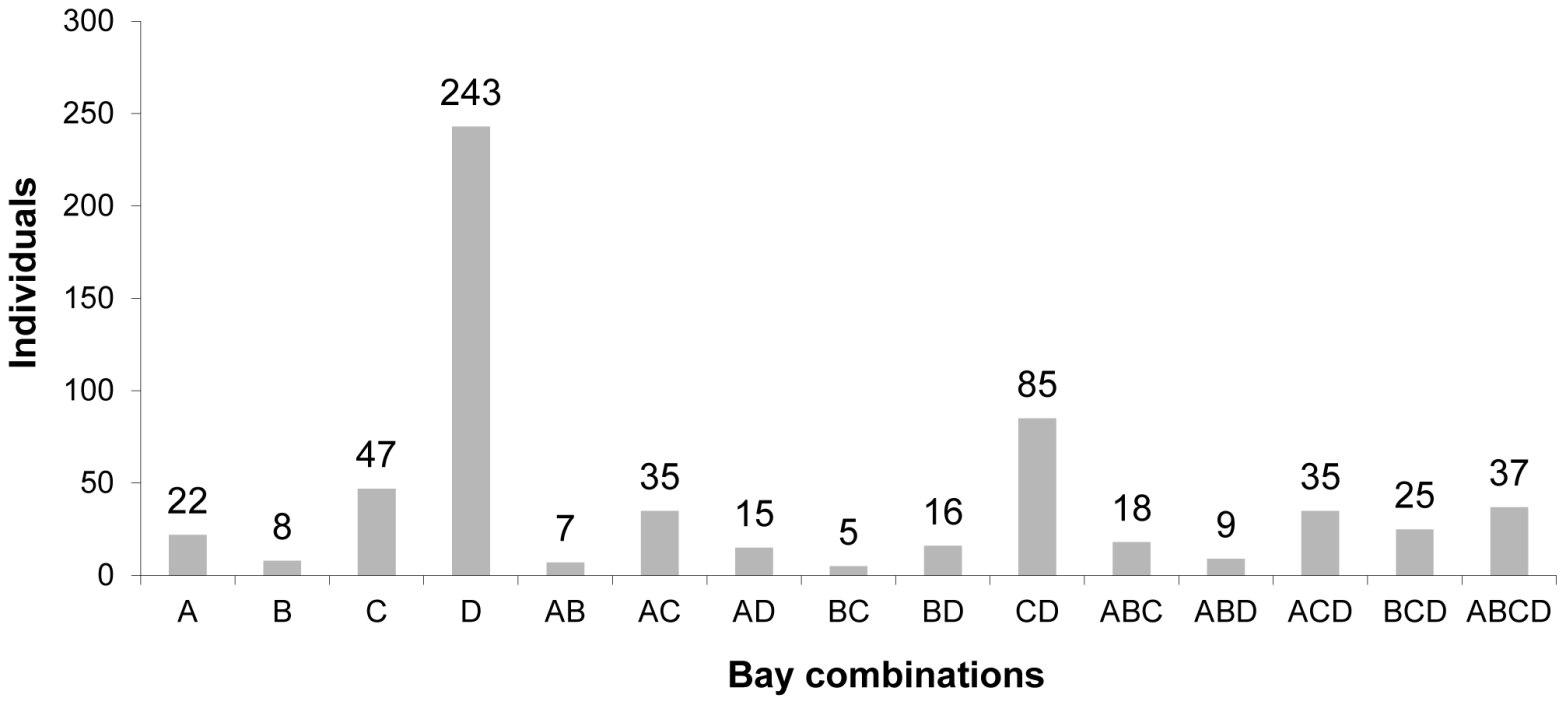
The combination of bays in which individual spinner dolphins have been sighted from September 2010 to August 2011. A = Kauhako Bay, B = Honaunau Bay, C = Kealakekua Bay and D = Makako Bay.

Seventy-six percent of individuals were photographed on more than one occasion, with one individual photographed as many as 18 times (Figure 2). On average, individual spinner dolphins were photographed on four (SE±0.14) occasions during the study period (Table 1). A cumulative discovery curve (Figure 3) indicated that the identification of new individuals was reaching a plateau before the end of the study period, with few new dolphins identified after 120 days of effort. Resting bay usage of individual spinner dolphins varied, in that some individuals were only photographed in one resting bay, while others were observed in all four resting bays (Figure 4).

**Table 1:** Number of photographic identification surveys, hours in each bay, spinner dolphin encounters in four resting bays along the Kona Coast of Hawai’i Island from September 2010 to August 2011.

Location	Surveys	Total surveyhours	Hours dolphinsabsent	Hours dolphins present	Hours photographing dolphins	Group encounter rate %	Mean group size	Min group size	Max group size
Kauhako Bay	44	407	257	150	28	39	29±3 SE	8	50
Honaunau Bay	22	195	116	79	16	41	24±4 SE	6	40
Kealakekua Bay	44	397	171	226	52	52	41±6 SE	5	110
Makako Bay	22	198	56	142	32	73	102±17 SE	25	250
Study Area	132	1197	600	597	128	49	49±6 SE	5	250

### Mark Rate of the Stock

To calculate the proportion of highly distinct individuals using the first independent measure, over 100,000 photographic images were randomly taken of spinner dolphins encountered in groups that comprised >20 individuals. Of these, 40,715 high-quality photographs contained distinctive and non-distinctive spinner dolphin dorsal fins. From the 40,715 high-quality photographs, 32,519 photographs contained distinctive individuals graded as *D1* or *D2*, and 14,405 were of highly distinctive individuals graded as *D1*. Therefore, the first independent measure estimating the proportion of identifiable individuals (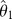) in the stock produced a mark rate of 35%:
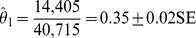



Of all the 65 groups encountered, a total of 14 groups comprised ≤20 dolphins. There were a total of 168 individual spinner dolphins encountered within these groups. Of these, 132 were distinctive individuals and 60 were highly distinctive *D1*. Thus, the second independent measure estimating the proportion of identifiable individuals (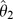) in the stock produced a mark rate of 36%:
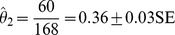



A Z-test showed that the two estimates were not significantly different (*p* = 0.68), the first value was used in all subsequent adjustments.

### Apparent Survival and Total Stock Abundance

The goodness of fit test to the open model did not suggest the presence of over-dispersion χ^2^ = 27, df = 26, p = 0.4275, and ĉ = χ^2^/df = 1.04. The abundance estimate of distinct individuals and a range of closed and open models are presented in Table 2. In all cases the estimates are very close to 214, the number of distinct animals seen, because the capture probabilities were very high (approx. 0.40 per period), consequently almost all animals were captured by the end of the study. This can also be seen by the flatness of the discovery curve (Figure 2). The closed and open models were very similar because we found that the annual apparent survival rate was 0.97±0.05 SE which is not significantly less than 1. As heterogeneity and time variation are strongly indicated for these data we used the estimate based on M_th_ 221±4.3 SE resulting in a total estimate of 631±60.1 SE (95% CI 524–761) spinner dolphins in the Hawai’i Island stock (Table 3).

**Table 2:** Highly distinctive (*D1*) population abundance estimates calculated from open and closed mark recapture models.

Open Model	Estimate of highlydistinctive individuals (*D1*)	Closed Models	Estimate of highly distinctive individuals (*D1*)
POPAN *φ* (*t*) *ρ*(*t*) *β*(*t*)	219±2.9 SE	M_0_	214±0 SE
		M_t_	214±0 SE
		M_h_ Jacknife	226±5.69 SE
		M_h_ Chao	221±4.32 SE
		M_th_ Chao	221±4.32 SE

Closed models: M_0_ = equal capture probability, M_t_ = variation in capture probability over time, M_h_ = individual heterogeneity of capture probability and M_th_ = variation in capture probability over time with individual heterogeneity of capture probability.

**Table 3:** Mark rates and abundance estimates from this study and previous studies.

Study	Mark rate	Markedindividuals	Total stock estimate
Thisstudy	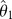 = 35% and 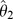 = 36%	214	631 (95% CI 524–761)
[6]	20%	192	960
[13]	29%	677	2,334
[14]	21.7% and 25.4%	217	1,001 and 855

## Discussion

This present study is the first concerted effort to estimate abundance and apparent survival rate estimates for the Hawai’i Island spinner dolphin stock. Two key conclusions can be drawn from this study that has implications for the management of this stock.

Firstly, our systematic sampling approach and capture/recapture analyses produced an apparent yearly survival estimate of 0.97±0.05 SE for this stock of spinner dolphins. Apparent survival represents the product of true survival and permanent emigration. Therefore, if permanent emigration approaches zero, apparent survival can be representative of true survival. The Hawai’i Island spinner dolphin stock is the most genetically distinct of the five island associated stocks [10], [12]. Therefore, permanent emigration of the Hawai’i Island stock could be assumed to be zero and consequently apparent survival is representative of true survival for the stock. Secondly, our total abundance estimate for this stock 631±60.1 SE (95% CI 524–761) is lower than any previous published estimates, 960 [6], 2,334 [13] and 855–1,001 [14] (Table 3).

The approaches employed by previous studies to collect photographic identification data to estimate abundance of Hawaiian spinner dolphins along the Kona Coast were not designed for specific capture-recapture models [6], [13], [14]. These previous studies used opportunistic photographic identification data retrospectively to estimate the population size. As a consequence, effects due to the inherent characteristics of individual spinner dolphins, and the variation in photographic identification effort throughout the study period weren’t allowed for. The proportion of distinctive individuals in these previous abundance estimates was determined by dividing the ‘*total number of identified individuals*’ by the ‘*mean percentage of individuals identified per group.*’ [6], [13], [14]. Therefore, the resulting abundance estimates did not take into account uncertainty or heterogeneity of individual capture probabilities and the estimates of the proportion of distinct animals were likely biased.

The systematic approach employed by this study was designed specifically to determine spinner dolphin abundance estimates using capture-recapture models. The consistent data collection effort throughout the study area and period (same bays on the same dates each month) helped to eliminate biases associated with the heterogeneity in capture probabilities due to the variation in individual characteristics. The use of only the highly distinct (*D1*) individuals helped to eliminate heterogeneity in capture probabilities due to variation in individual distinctiveness, and furthermore reduced misidentification errors of individuals during the identification process. Two independent methods used to determine the proportion of distinctive individuals produced similar results (∼36%). These proportions are higher than reported in previous studies in the region [6], [13], [14]. Advances in digital imaging technology allowed for a greater number of spinner dolphin images to be taken, compared to previous studies that relied on film photography and processing [5], [6], [13], [14]. Furthermore, advances in technology allowed for high resolution dolphin images, which, in turn, allowed less-distinctive individuals to be identified and included in the catalogue, more so than in previous studies that may have categorised the same quality of photographs as non-distinctive, and as a consequence may have contributed to the low proportion of distinctive individuals identified (Table 3).

As our study lasted for one year we expected that we would need to use an open capture-recapture model. However, we found that closed population models gave almost identical population estimates to the open models. We think the population is approximately closed for two reasons. First, the Hawai’i Island spinner dolphin stock is genetically distinct from the other island associated spinner dolphin stocks in the Hawaiian archipelago [10], which is strong evidence for there being little movement in or out of this area. In fact, evidence from recent genetic work indicates that spinner dolphins inhabiting the Kona Coast of Hawai’i Island exhibit a greater degree of philopatry than any other spinner dolphin stock in the Main Hawaiian Islands [10]. Second, spinner dolphins are long-lived animals with an estimated annual survival rate of 0.97±0.05 SE and even though a shift in spinner dolphin sighting distribution from the leeward side to the windward side of Hawai’i Island has been documented [6], this shift was for only one month (a lot shorter than our sampling period), after which it shifted back to the leeward side [6] and would be included in our closed population estimate. Therefore no significant new recruits would be expected to enter the population in just one year. As heterogeneity and time variation of capture probabilities are strongly suggested we decided to use the abundance estimate based on the closed model M_th._ However, in this case the estimates of all the models are almost identical (Table 3).

Bias can also be introduced into abundance estimates from misidentification of individuals. This can occur in two ways: one individual being identified as two individuals (positive bias) and two individuals being identified as one individual (negative bias). In this study only highly distinctive spinner dolphins were used which helps to mitigate the introduction of bias from individual misidentification.

The photographic identification of individuals for survival rates and abundance estimates was undertaken across the four major resting bays along the Kona Coast but did not survey the entire coastline of Hawai’i Island. It is possible that the abundance estimate of this stock underestimates the whole Kona Coast spinner dolphin population. However, we suspect that any potential underestimation is insignificant and that almost all members of the population use these four main resting bays. Earlier studies documented spinner dolphins on the windward side of Hawai’i Island [6], however, it is unlikely that this represents prime resting habitat for them given results on habitat preference studies [17]. Earlier studies also observed individual spinner dolphins moving from the north to the south of the Kona Coast encompassing the four main resting bays of this study [6], [13], [14]. In addition, radio tagged individual spinner dolphins have been observed travelling 20–70 km along the Kona Coast [6]. This study is part of a larger project (SAPPHIRE) in which spinner dolphin group focal follows were also undertaken outside, and to the north and south of the four resting bays. Individual spinner dolphins observed during these focal follows were also observed in at least one of the four main resting bays during our photographic identification (Tyne, J.A, unpublished data). This suggests our work sampled the entire population of the Hawai’i Island stock [10], [12].

### Management Implications

The dolphin-watch tourism industry in Kona has increased over the past 20 years [56], paralleling the dramatic increase in the industry worldwide [57]. Recent short-term research has suggested that an increase in human traffic inside and outside of the dolphin resting habitats [14], [19], [21] resulted in dolphins spending less time in these resting habitats (e.g. [14]) and that their resting behaviour was interrupted as a consequence. It has been suggested that spinner dolphins may leave the bays in direct response to human interactions [19]–[21], [28]. However, it was not possible to identify population level effects from these short-term studies. Elsewhere, dolphin-human interactions have had detrimental effects on the focal population. In New Zealand, the resting behaviour of bottlenose dolphins decreased as the number of boats increased in the Bay of Islands [58] and in Milford Sound [59]. In Shark Bay, Western Australia, long-term exposure to dolphin-watch vessels caused declines in relative abundance of bottlenose dolphins in an area where boat-based tourism occurred [26].

Due to growing concerns, the United States National Oceanic and Atmospheric Administration’s Fisheries Service Pacific Islands Regional Office, in conjunction with the Pacific Islands Fisheries Science Centre, published a Notice of Intent [60] to prepare an Environmental Impact Statement assessing potential impacts of a proposed rulemaking on human activity. The proposed rule seeks to implement time-area closures in specific spinner dolphin resting habitat to reduce the cumulative exposure to human activity along the Kona Coast of Hawai’i Island [60].

The rigorous systematic sampling during this study produced the first baseline estimates of abundance and apparent survival rates for the Hawai’i Island spinner dolphin stock. These estimates can provide valuable assistance to management agencies, for comparison with historical estimates and to assess the effectiveness of future management actions seeking to mitigate negative human-dolphin interactions. The current estimate of 631 (95% CI 524–761) is substantially lower than previous abundance estimates (Table 3). When this estimate is combined with the rigid daily behavioural pattern of spinner dolphins, the genetic distinctiveness of the stock and the ease of human access to the spinner dolphins in their preferred resting habitats, this stock is likely more vulnerable to negative impacts from human disturbance than previously believed.
